# Assessment of the Effects of the Synbiotic Combination of *Bifidobacterium longum* subsp. *infantis* CECT 7210 and Oligofructose-Enriched Inulin Against Digestive Bacterial Infections in a Piglet Model

**DOI:** 10.3389/fmicb.2022.831737

**Published:** 2022-03-08

**Authors:** Agustina Rodríguez-Sorrento, Lorena Castillejos, Paola López-Colom, Gloria Cifuentes-Orjuela, José A. Moreno-Muñoz, Susana M. Martín-Orúe

**Affiliations:** ^1^Servicio de Nutrición y Bienestar Animal, Departament de Ciència Animal i Dels Aliments, Universitat Autònoma de Barcelona, Bellaterra, Spain; ^2^Departamento de Producción Animal, Facultad de Medicina Veterinaria y Zootecnia, Universidad Agraria del Ecuador (UAE), Guayaquil, Ecuador; ^3^Laboratorios Ordesa SL, Parc Científic de Barcelona, Barcelona, Spain

**Keywords:** probiotic, piglet, ETEC, *Salmonella*, fructo oligosaccharides, prebiotic, inulin, bifidobacteria

## Abstract

The use of bifidobacteria as probiotics has proven to be beneficial in gastroenteric infections. Furthermore, prebiotics such as inulin can enhance the survival and growth of these bacteria. Two trials were performed to evaluate the effects of the administration of *Bifidobacterium longum* subsp. *infantis* CECT 7210 and oligofructose-enriched inulin against *Salmonella enterica* serovar Typhimurium or enterotoxigenic *Escherichia coli* (ETEC) F4. A total of 72 (*Salmonella* trial) and 96 (ETEC F4 trial) weaned piglets were used in a 2 × 2 design (with or without synbiotic, inoculated or not with the pathogen). After adaptation, animals were orally inoculated. Performance and clinical signs were evaluated. On days 4 and 8 (*Salmonella* trial) and 3 and 7 (ETEC F4 trial) post-inoculation (PI), one animal per pen was euthanized. Blood, digestive content and tissue samples were collected and microbiological counts, fermentation products, serum inflammatory markers and ileum histomorphometry analysis were performed. Both challenges had an impact on faecal consistency (*p* < 0.001), including the faecal shedding of *Salmonella* and increased numbers of enterobacteria and coliforms. The synbiotic administration did not have any effect on pathogen loads but induced changes in the fermentation profile, such as increased valeric acid in both trials as well as decreased acetic acid, except for *Salmonella*-challenged animals. The effect on propionate varied among trials, increasing in challenged synbiotic-treated pigs and decreasing in non-challenged ones in the *Salmonella* trial (P interaction = 0.013), while the opposed occurred in the ETEC F4 trial (P interaction = 0.013). The administration of the synbiotic increased intraepithelial lymphocytes (IEL; *p* = 0.039) on day 8 PI in the *Salmonella* trial and a similar trend occurred in non-challenged pigs in the ETEC F4 trial (P interaction = 0.086). The results did not provide evidence of reduced pathogen load with the synbiotic, although a modulation in fermentative activity could be identified depending on the challenge. Consistent increases were found in IEL, suggesting that this synbiotic combination has some immunomodulatory properties.

## Introduction

Acute enteritis is a pathology that consists of a loss of faecal consistency and/or increased stool frequency, with a duration of less than 7 days. In the vast majority of cases, it is precipitated by an infectious agent, this being a frequent cause of global childhood mortality and morbidity, especially in the developing world (Thapar and Sanderson, 2004). The list of organisms that can provoke acute infectious enteritis includes viruses, bacteria and parasites. As many as 70% of cases are generated by a virus (principally rotavirus), while a non-negligible 20% are caused by a bacterial infection (Koletzko and Osterrieder, 2009). Among these bacteria, *Salmonella* and *Escherichia coli* can often be isolated, with a total of 25,708 (20, 385 *Salmonella*; 5, 323 *E. coli*) cases reported to the National Outbreak Reporting System in the United States between 2015 and 2018 (CDC, 2021). These infections commonly have their origin in food contamination, although the infection can also be produced *via* contact with infected animals (Conrad et al., 2017). New-born and young children are particularly susceptible to infections because their immune systems are not mature and they are not fully immunocompetent. An important form of protection is provided by the mother through passive IgG transplacental transfer, as well as in the milk (Simon et al., 2015) as it has been demonstrated that breastfeeding diminishes the incidence and severity of infectious diarrhoea (Farthing et al., 2013; Hartman et al., 2019).

Probiotics are live beneficial microorganisms that, when administered in infant formulas, can help reduce the number of episodes and duration of diarrhoea associated with acute infections (Szajewska and Mrukowicz, 2001). Nonetheless, this effect is strain-dependent and different outcomes have been reported in the literature (Skórka et al., 2017). *Bifidobacterium longum* subsp. *infantis* CECT 7210 is a bifidobacteria isolated from infant faeces that when given as a supplement to healthy children, has been associated with a reduction in diarrhoea events (Escribano et al., 2018). Moreover, by the use of different animal models, previous results have evidenced the ability of this strain to provide protection against rotavirus infection using murine models (Moreno-Muñoz et al., 2011) and reduce *Salmonella* shedding in oral challenged piglets (Barba-Vidal et al., 2017). Prebiotics such as inulin or its derivatives can potentially benefit the survival and multiplication of bifidobacteria (Vandeputte et al., 2017) and combat enteric pathogens (Tran et al., 2018).

Therefore, it is hypothesised that the synbiotic administration of an advantageous *Bifidobacterium* strain with these prebiotics will result in an improved outcome regarding digestive bacterial illness. The aim of this work is to determine the efficacy of a synbiotic combination of *B. longum* subsp. *infantis* CECT 7210 and inulin enriched with oligofructose against *Salmonella* Typhimurium and enterotoxigenic *E. coli* F4, using weaned piglets as an animal model.

## Materials and Methods

Two different experiments were executed to evaluate the efficacy of the synbiotic combination against an oral challenge with either *Salmonella enterica* serovar Typhimurium (*Salmonella* trial) or enterotoxigenic *E. coli* F4 (ETEC F4 trial). Both experiments were performed at the Experimental Unit of the Universitat Autònoma de Barcelona (UAB) and received prior approval (Permit No. CEEAH: 4026 DMAH: 10118) from the Animal and Human Experimental Ethical Committee of this institution and its competent authorities. The treatment, management, housing, husbandry and slaughtering conditions conformed to European Union Guidelines (Directive 2010/63/EU*, European Commission, 2010*). All efforts were made to minimise animal suffering.

### Animals, Housing and Experimental Design

These trials were carried out as biosafety Level 2 procedures and all personnel involved received appropriate training. A total of 168 male piglets were distributed between the two trials as follows: 72 (Landrace × Large White) × Pietrain of 24 (± 4) days of age weighing 7.70 (± 0.15) kg for the *Salmonella* trial and 96 (Landrace × Large White) × Pietrain piglets of 21 (± 4) days of age weighing 4.98 (± 0.07) kg for the ETEC F4 trial. All animals came from high-sanitary-status farms and mothers that were serologically negative to *Salmonella* in the *Salmonella* trial or were not vaccinated against *E. coli* in the ETEC F4 trial.

Piglets were transported to the experimental unit located at the Autonomous University of Barcelona, comprising three boxes (*Salmonella* trial) and four boxes (ETEC F4 trial) of eight pens each (24 and 32 pens, respectively, with three animals per pen). Each 2m^2^ pen was separated by a solid fence that prevented any contact between animals of different pens. Each pen had a feeder and water nipple to provide feed and water *ad libitum*. All weaning rooms were equipped with an automatic heater and forced ventilation and each pen had an individual heating light.

At arrival, the animals were distributed according to their initial body weight (BW) in order to ensure a homogeneous average body weight between treatment groups. Trials consisted of a factorial design 2 × 2 that included two treatments (control vs. synbiotic) and challenged or not with the pathogen (yes vs. no), resulting in a total of four experimental groups: control non-inoculated animals, NC; non-inoculated animals receiving synbiotic treatment, NS; control inoculated animals, IC; and inoculated animals receiving synbiotic treatment, IS. In the case of the first trial, the design was unbalanced, as piglets in two of the three rooms were challenged while the third room remained non-challenged. In contrast, for the second trial, we utilised four rooms and therefore had a balanced design. In each room, synbiotic treatment was distributed within four pens on one side of the room, with the four control pens on the other side of the room separated by a corridor to prevent contact between animals. Each experimental group had eight replicates, except for the non-challenged groups in the *Salmonella* trial, which had four replicates instead.

### Probiotic Strains, Prebiotic and Diets

In both trials, the tested probiotic was *B. longum* subsp. *infantis* CECT 7210 strain, supplied by Ordesa SL in a lyophilized form and containing 5 × 10^10^ colony-forming units (cfu) per gram of product in a maltodextrin carrier. The estimated dosage during the procedures was the same for both trials (1 × 10^9^ cfu per piglet and day). In the *Salmonella* trial, the animals received the probiotic orally each morning using disposable syringes without a needle. To this end, the lyophilized bacteria were re-suspended in 2 ml of phosphate-buffered saline (PBS) no more than 1 h prior to administration. The control groups were administered the same amount of sterile PBS as a placebo. In the ETEC F4 trial, the piglets received the probiotic mixed in their feed with the aim of simplifying daily work and minimise the manipulation and the stress of the animals. Each day, the lyophilized probiotic was thoroughly mixed manually with fresh feed, with the dose adjusted considering the average feed intake (1 g of lyophilized probiotic per 1,000 g of feed). The stability of the probiotic into the feed and feeders was previously assessed in a viability test to ensure an accurate dosage of the product per day.

The prebiotic consisted of a mixture of oligofructose (FOS) and inulin (Orafti^®^ Synergy1, Beneo; Mannheim, Germany) that was administered through the feed (5%) in both trials.

Pre-starter diets were formulated in concordance with the nutrient requirement standards for pigs (NRC, 2012) and given in a mash form. In the *Salmonella* trial, both experimental diets were manufactured from the same single basal batch (with the same formula) being the prebiotic subsequently mixed with half of the batch. In the ETEC F4 trial, potential amino acid dilution in the synbiotic diet due to the incorporation of the prebiotic was compensated by the addition of synthetic amino acids with different adjusted formulae for each experimental diet (synbiotic diet included as: 0.5 g L-valine, 0.9 g L-lysine HCL, 1.2 g DL-methionine, 0.5 g L-threonine and 0.2 g L-tryptophan per kg of feed); in this way, we could guarantee the same level of the most limiting amino acids in both experimental diets. Details of the ingredient and chemical composition are given in Table 1.

**Table 1:** Ingredient and nutritional composition of the diets.

Ingredients (g/kg FM)	*Salmonella* Trial		ETEC F4 Trial	
	Control	Synbiotic	Control	Synbiotic
Maize	280.8	266.8	207.4	196.3
Wheat	170.0	161.5	180.0	170.0
Barley 2 row	150.0	142.5	170.0	160.9
Extruded soybean	122.4	116.3	149.1	141.1
Sweet whey powder (cattle)	100.0	95.0	100.0	94.6
Fish meal	50.0	47.5	60.0	56.8
Soybean meal 44	50.0	47.5	80.0	75.7
Whey powder 50% fat	30.3	28.8	25.0	23.6
Mono-calcium phosphate	21.3	20.2	6.8	6.4
Calcium carbonate (CaCO_3_)	8.2	7.8	3.9	3.6
L-Lysine HCL	4.5	4.3	4.5	5.0
Vit-Min Premix^*^	4.0	3.8	4.0	3.7
Sodium chloride (marine salt)	3.0	2.9	2.5	2.3
DL-Methionine 99	2.4	2.3	2.6	3.6
L-Threonine	2.3	2.2	2.3	2.6
L-Tryptophan	0.9	0.9	0.6	0.7
L-Valine	1.0	1.0	1.5	1.9
Prebiotic	0	50	0	50
Analysed composition (g/kg FM)	Control	Synbiotic	Control	Synbiotic
Dry matter	903.4	902.9	909.6	912.7
Ashes	68.5	61.6	52.9	50.4
Crude fat	57.7	53.8	60.2	56.1
Crude protein	174.0	161.4	204.7	180.7
Neutral detergent fibre	89.8	119.2	92.2	83.0
Acid-detergent fibre	29.4	27.3	30.3	29.4

* Provided per kilogram of complete diet: 10, 200 IU vitamin A, 2,100 IU vitamin D3, 39.9 mg vitamin E, 3 mg vitamin K3, 2 mg vitamin B1, 2.3 mg vitamin B2, 3 mg vitamin B6, 0.025 mg vitamin B12, 20 mg calcium pantothenate, 60 mg nicotinic acid, 0.1 mg biotin, 0.5 mg folic acid, 150 mg Fe, 156 mg Cu, 0.5 mg Co, 120 mg Zn, 49.8 mg Mn, 2 mg I, and 0.3 mg Se.

### *Salmonella* and *ETEC* Strains

In the first trial, the bacterial strain used for the oral challenge was a *Salmonella enterica* serovar Typhimurium var. monophasic (*formula: 4,5,12:i:-, resistance profile: ACSSuT-Ge, Fagotype: U302*) that had been isolated from a salmonellosis outbreak of fattening pigs in Spain, provided by the Infectious Diseases Laboratory (Ref. 301/99) of the UAB. Preparation of the oral inoculum consisted of 24-h incubation at 37°C in buffered peptone water (BPW; Oxoid; Hampshire, United Kingdom) and diluted (1:10) with sterile PBS (Sigma-Aldrich; Madrid, Spain). The final concentration of the inoculum was 1 × 10^9^ cfu/ml. Inoculum concentrations were determined prior to the inoculation by McFarland standards and were doubly plated in Tryptic Soy Agar (Liofilche; Italy) on the same day in order that they could be checked by manual plate counting.

In the second trial, the bacterial strain of enterotoxigenic *E. coli* F4 used was isolated from the faeces of 14-week-old pigs and provided by the Infectious Diseases Laboratory (Ref. 30/14) of the UAB. This strain presented the following virulence factors: F4ab, F4ac, LT, STb and EAST1 and was negative for K99, F6, F18, F41, STa, VT1 and VT2 y EAE. The oral inoculum was prepared *via* 12-h overnight incubation at 37°C in Brain Heart Infusion broth (Oxoid; Hampshire, England) with slow agitation (250 rpm) in an orbital incubator. The culture was directly given to the animals with a final concentration of 1·10^9^ cfu/ml. Inoculum concentrations were also determined before the inoculation by McFarland standards and were plated in Luria Agar (LA; made in-house: tryptase, yeast extract, NaCl, agar and Oxoid; Hampshire, UK) the same day for manual plate counting.

### Experimental Procedure

Both experiments lasted 15 days. One animal from each pen was euthanized on days 4 and 8 post-inoculation (PI) in the *Salmonella* trial and on days 3 and 7 PI in the ETEC F4 trial.

After an adaptation period of 7 days in the *Salmonella* trial and 8 days in the ETEC F4 trial, an inoculum containing the pathogenic bacteria culture was given to the challenged groups orally: in the first trial, this was one 2 ml dose (2 × 10^9^ cfu) of *Salmonella* Typhimurium, whereas in the second trial, it was one 6 ml dose (6 × 10^9^ cfu) of ETEC F4. The same amount of sterile broth was administered to the non-challenged piglets. In order to ensure that the animals’ stomachs were full at the time of the oral challenge, pigs were starved for a period of 12 h and feed was reintroduced 30 min before inoculation.

From the challenge onwards, the animals’ clinical signs were checked daily to evaluate their post-inoculation status (i.e. dehydration, anorexia, apathy, general behaviour and faecal score), always by the same person. Faecal score was measured using a scale whereby 1 = solid and cloddy, 2 = soft with shape, 3 = very soft or viscous liquid and 4 = watery or with blood. Rectal temperature was assessed using a digital thermometer (Accuvet, Sanchung City, Taiwan) on days 1, 2 and 3 PI in the *Salmonella* trial and days 1 and 2 PI in the ETEC F4 trial.

The animals’ performance was also monitored as: individual body weight (BW) was registered on arrival and on days 0, 4 and 8 PI (0, 3 and 7 PI in the ETEC F4 trial) and feed intake was determined on days 0, 4 and 8 PI in the *Salmonella* trial, whereas in the ETEC F4 trial, feed intake was registered daily, concurring with the regular feed replacement aimed at maintaining probiotic viability. The average daily gain (ADG), average daily feed intake (ADFI) and the gain:feed ratio (G:F) were calculated by pen. The mortality rate was also registered and no antibiotic treatment was given to the animals in any of the experiments.

For microbiological analysis, faecal samples were collected aseptically after spontaneous defecation or by digital stimulation at arrival on the day of the inoculation (0 PI): in the *Salmonella* trial, this was from the animal with the highest initial BW in each pen (*N* = 24), whereas in the ETEC F4 trial, faecal samples were obtained from the animal with the medium BW in each pen (*N* = 32). Furthermore (and just for the *Salmonella* trial), additional faecal samples were taken on days 1, 3 and 7 PI from the same animal.

On days 4 and 8 PI (3 and 7 PI in the ETEC F4 trial), one pig per pen was euthanized. Slight differences in sampling days between trials were due to logistic reasons. On day 4 PI, the animal selected was the one with the medium initial BW, while on day 8 PI, it was the heaviest piglet in each pen. The animals were euthanized and sequentially sampled during the morning of each day (between 8:00 and 13:00 h). Before injecting the euthanasia drug, 10 ml sample blood was taken from each animal *via* venepuncture of the cranial cava vein using 10 ml blood collection tubes without anticoagulant (Aquisel; Madrid, Spain). Immediately after blood sampling, pigs were intravenously administered a lethal dose injection of sodium pentobarbital (140 mg/kg BW; Euthasol, Le Vet B.V.; Oudewater, Netherlands). Once dead, the animals were bled, the abdomen opened and the gastrointestinal tract extracted.

A faecal sample from the rectum was used for traditional microbiology in the ETEC F4 trial, whereas a caecal sample was obtained for microbiology in the *Salmonella* trial. They were kept on ice and analysed within 4 hours.

In both experiments, the digesta of the ileum and the proximal colon were collected and homogenised prior to pH determination with a pH meter calibrated on each day of use (Crison 52–32 electrode, Net Interlab; Barcelona, Spain) and the digesta score was registered on a scale as follows: 1 = liquid; 2 = liquid with some formed material; 3 = thick; and 4 = semi-solid. Subsamples of the ileal and colonic digesta were preserved for different analyses. One aliquot of colonic content was kept at −80°C for ETEC F4 (ETEC F4 trial) and probiotic quantification by qPCR. A set of ileal and colonic digesta samples were conserved frozen at −20°C in H_2_SO_4_ solution (3 ml of content plus 3 ml of 0.2 N H_2_SO_4_) for ammonia (NH_3_) determination and an additional set (~10 g) was also frozen (−20°C) for future analysis of short-chain fatty acids (SCFA) and lactic acid.

In the ETEC F4 trial, to determine the number of enterobacteria, coliforms and ETEC F4 attached to the intestinal mucosa, 5 cm sections of distal ileum were collected from each animal, washed thoroughly with sterile PBS, opened longitudinally and scraped with a microscopy glass slide to obtain the mucosa scraping.

For the histological study, 1 cm sections from the ileum were removed, opened longitudinally, thoroughly and carefully washed with 4% formaldehyde solution (Panreac; Castellar del Vallès, Spain) and fixed by immersion in the same solution.

Blood samples were centrifuged (3,000 × g for 15 min at 4°C) after clotting and the serum obtained was stored at −20°C.

### Analytical Procedures

#### Feed Analysis

Chemical analyses of the diets, including dry matter (DM), ash, crude protein and diethyl ether extract, were performed according to Association of Official Agricultural Chemists standard procedures (AOAC, 1995). Neutral detergent fibre and acid-detergent fibre were determined according to the method of Van Soest et al. (1991).

#### Microbiological Analysis

For the microbiological analysis of *Salmonella*, samples were transferred to buffered peptone water solution in a concentration of 1:10. The quantitative analysis was performed by seeding serial dilutions of the samples 10^−2^, 10^−4^ and 10^−6^ in Xylose-Lactose-Tergitol-4 plates (XLT-4; Merck; Madrid, Spain). For the qualitative analysis, samples were incubated in BPW (37°C, 24 h), transferring 100 μl of the culture to 10 ml of Rappaport-Vassiliadis for a second incubation (42°C, 48 h) to finally seed them in XLT4 plates to observe H_2_S positive colonies.

For the enterobacteria and coliform counts, samples were serially diluted in Lactate Ringer Solution (Sigma-Aldrich; Madrid, Spain) and proper dilutions seeded in MacConkey agar (Oxoid; Madrid, Spain) and eosin methylene blue agar (Scharlab; Barcelona, Spain). Plaques were incubated for 24 h at 37°C and colonies were manually counted.

The presence of ETEC F4 in the colonic digesta and ileal scrapings was determined by real-time PCR. To extract the DNA from these samples, the commercial QIAmp DNA stool minikit (Qiagen; West Sussex, United Kingdom) was utilised. Afterwards, several aliquots of DNA eluted in Qiagen buffer AE (total volume; 200 μl) were stored frozen at −80°C. A qPCR targeting the gene coding the F4 fimbria of ETEC F4 using the SYBR green dye was performed according to the procedure described by Hermes et al. (2013). To express the results, the animals were distributed across five levels according to the number of gene copies per gram of fresh matter that they showed when qPCR was performed. Ranges were defined as follows: negative = under 4 logarithmic units of gene copies per gram of fresh matter; low = 4–5.5 logarithmic units of gene copies per gram of fresh matter; medium = 5.5–7 logarithmic units of gene copies per gram of fresh matter; high = 7–8.5 logarithmic units of gene copies per gram of fresh matter; and very high = more than 8.5 logarithmic units of gene copies per gram of fresh matter.

#### Short-Chain Fatty Acids, Lactic Acid and Ammonia Analyses

Short-chain fatty acids and lactic acid analyses were performed using gas chromatography, after the samples had undergone acid–base treatment followed by ether extraction and derivatization with N-(tertbutyldimethylsilyl)-N-methyl-trifluoroacetamide plus 1% tert-butyldimethylchlorosilane agent, using the method of Richardson et al. (1989) that was subsequently modified by Jensen et al. (1995).

Ammonia concentrations were assessed using a gas-sensitive electrode (Hatch Co.; Colorado, United States) combined with a digital voltmeter (Crison GLP 22, Crison Instruments, SA; Barcelona, Spain), following a procedure described by Hermes et al. (2009) that was adapted from Diebold et al. (2004). Samples were diluted (1:2) in 0.16 M NaOH and, after homogenisation, were centrifuged at 1500 × g for 10 min. Once the ammonia was released, it was measured in the supernatants as a change in voltage in mV.

#### Serum Analysis

Serum concentrations of Tumour Necrosis Factor-α (TNF-α) were determined by Quantikine Porcine TNF-α kits (R&D Systems; Minneapolis, United States) and pig major acute-phase protein (Pig-MAP) concentration was determined by a sandwich-type enzyme-linked immunosorbent assay (ELISA; Pig MAP Kit ELISA, Pig CHAMP Pro Europe SA; Segovia, Spain) according to the manufacturer’s instructions. In the *Salmonella* trial, antibodies against *Salmonella* were also assessed using an ELISA *Salmonella* Herdcheck (Idexx; Hoofddorp, Netherlands), establishing the cut-off for positivity in optic density ≥ 40%.

#### Histological Analysis

For histological study, tissue samples were dehydrated and embedded in paraffin wax, sectioned at a thickness of 4 μm and stained with haematoxylin and eosin. The measurements of 10 different villus-crypt complexes per sample and the counting of intraepithelial lymphocytes (IEL), goblet cells (GC) and the number of mitosis of each were performed with a light microscope (BHS, Olympus; Barcelona Spain), using as a guideline the procedure described in Nofrarías et al. (2006). Briefly, the villus height and crypt depth were measured using a linear ocular micrometre (Olympus, Microplanet). The same villus and crypt columns were used to determine the number of IEL, GC and mitosis. On the basis of the cellular morphology, differences between the nuclei of enterocytes, mitotic figures, goblet cells and lymphocytes were clearly distinguishable at 400× magnification. All morphometric analysis was done by the same person, who was blinded to the treatments.

### Statistical Analysis

The results are expressed as means with their standard errors unless otherwise stated. Microbiological counts were log transformed for analysis. A two-way analysis of variance (ANOVA) was used to examine the effect of the two experimental treatments and the inoculation with the following model:


$$Y_{ijk}=m+{\mathrm{treat}}_{i}+{\mathrm{challenge}}_{j}+\left({\mathrm{treat}}^{*}{\mathrm{challenge}}_{ij}\right)+e_{ij}$$

where Y_ijk_ relates to each observation of the outcome variable, m is the global mean, treat_i_ is the main effect of treatment, challenge_j_ is the main effect of the oral pathogen challenge and treat^*^challenge_ij_ corresponds to the interaction between treatment and the challenge. Finally, e_ij_ is the experimental error term. Regarding the interaction term, it was removed from the model when found not to be significant.

The effects on the post-slaughter measurements were examined using the R v3.4 (R Core Team, 2013) lm function for two-way ANOVA, with the treatment and the challenge effects as main effects. For *Salmonella* and *E. coli* F4 prevalence values and Pig-MAP serum concentrations, data were subjected to frequency analysis using the fisher. Test function in the same package.

ADFI and daily faecal scores were also analysed using the lme4 package (Bates et al., 2015) lmer function for a generalised linear mixed model with a treatment-by-time interaction term and considering the animal as the random effect.

The experimental unit was the pen. For all analyses, significance was set at *p* < 0.05 and values of *p* ≥0.05 but ≤0.10 were considered to indicate a statistical trend. When treatment effects were established, the mean comparison was adjusted with the Tukey–Kramer test. Data are presented as means and residual standard deviation.

## Results

Both experiments proceeded as expected, without any remarkable incidence.

The oral challenge with the pathogenic bacteria induced moderate clinical signs in the animals that were slightly more severe after the *Salmonella* challenge (*Salmonella* trial). In these trials, humane euthanasia of two pigs was indicated (1 IS; 1 NIS). Furthermore, two spontaneous casualties were registered in the *Salmonella* trial (1 IC; 1 IS) and the ETEC F4 trial (1 NIS; 1 IC).

### Performance Parameters

Changes in ADG, ADFI and gain:feed ratio (G:F) with the experimental treatments are shown in Table 2.

**Table 2:** Effects of experimental treatments on feed intake and weight gain.

	Treatment				RSD	*p*-value	
	IC	IS	NIC	NIS		Challenge	Treatment
*Salmonella* trial BW (kg)							
Initial	7.70	7.74	7.70	7.66	*0.156*	*0.540*	*0.878*
Final	9.23	9.71	10.52	10.26	*1.311*	*0.120*	*0.662*
ADFI (g)							
pre	181.1	189.1	192.1	214.5	*43.66*	*0.347*	*0.480*
post	288.2	287.4	398.7	403.2	*93.08*	0.010^*^	*0.979*
ADG (g)							
pre	121.6	89.4	126.1	111.1	*42.85*	*0.146*	*0.488*
post	85.2	109.2	249.9	165.9	*104.90*	0.024^*^	*0.781*
G:F							
pre	0.67	0.44	0.66	0.52	*0.197*	*0.704*	0.020^*^
post	0.26	0.33	0.60	0.36	*0.268*	*0.125*	*0.737*
ETEC F4 trial BW (kg)							
Initial	4.99	5.02	4.97	4.96	*0.790*	*0.107*	*0.723*
Final	7.46	6.94	7.20	7.14	*0.775*	*0.915*	*0.290*
ADFI (g)							
pre	82.8	77.5	98.2	79.2	*17.03*	*0.506*	*0.215*
post	359.3	308.0	370.5	365.4	*65.95*	*0.152*	*0.237*
ADG (g)							
pre	55.5	57.3	69.1	48.7	*32.30*	*0.830*	*0.421*
post	283.2	219.2	250.5	235.2	*72.01*	*0.745*	*0.130*
G:F							
pre	0.61	0.73	0.76	0.53	*0.349*	*0.853*	*0.674*
post	0.79	0.70	0.68	0.63	*0.152*	*0.102*	*0.207*

Body weight (BW; kg), average daily feed intake (ADFI; g/day), average daily gain (ADG; g/day) and feed efficiency (gain:feed ratio, G:F) for the pre-inoculation period (pre: days 1–8 Salmonella trial and days 1–9 ETEC trial) and post-inoculation period (post: days 8–15 Salmonella trial and days 9–15 ETEC trial). IC, Inoculated animals receiving placebo; IS, Inoculated animals receiving the synbiotic; NIC, Non-inoculated animals receiving placebo; and NIS, Non-inoculated animals receiving the synbiotic. *N* = 8 for all groups except for non-challenged animals in Salmonella trial, *N* = 4. No interaction effects between challenge and treatment were found. RSD, residual standard deviation. *indicates statistical difference.

The challenge with the pathogen caused a decrease in ADFI and ADG in the *Salmonella* trial (*p* = 0.010 and *p* = 0.024, respectively). The effects of the ETEC F4 challenge were milder, with only a numerical trend seen for a lower ADFI in the post-inoculation period (333.65 vs. 367.95 g for challenged and non-challenged groups, respectively, *p* = 0.152).

No significant changes in ADFI nor ADG were registered that might be associated with the synbiotic treatment regardless of the trial, aside from a numerical difference (*p* = 0.130) in the ETEC F4 trial for reduced ADG during the post-inoculation phase. Moreover, in the *Salmonella* trial, a reduction in the G:F ratio was seen after the adaptation period (*p* = 0.020).

### Clinical Signs

On day 1 PI, an increment of 1°C of rectal temperature was caused by the *Salmonella* challenge (38.9 ± 0.13°C vs. 39.8 ± 0.18°C, *p* < 0.001), whereas the ETEC F4 challenge did not alter the piglets’ temperatures (39.1 ± 0.07°C vs. 39.1 ± 0.17°C, *p* = 0.974). No significant differences were found related to the synbiotic administration.

**Figure 1:**
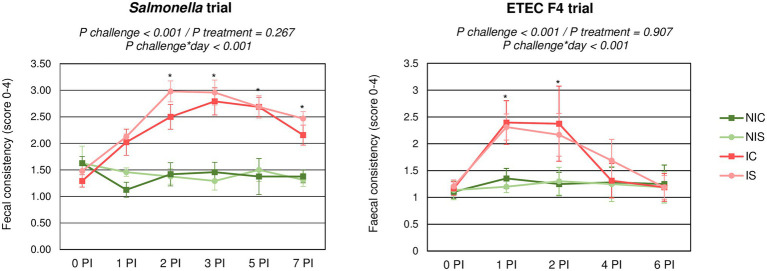
Evolution of average faecal scores for the different experimental groups during the post-inoculation (PI) period. IC, Inoculated animals receiving placebo; IS, Inoculated animals receiving the synbiotic; NIC, Non-inoculated animals receiving placebo; and NIS, Non-inoculated animals receiving the synbiotic. *N* = 8 for all groups except for non-challenged animals in *Salmonella* trial, *N* = 4. Bars correspond to standard error. *indicates statistical difference.

Figure 1 shows the evolution in faecal consistency after the oral challenge for each trial. In both trials, the challenge was able to significantly impair faecal consistency (*p* < 0.001) with an increase in the incidence of diarrhoea. However, the progression of the faecal score over time differed between the trials: whereas with the *Salmonella* challenge, the impairment of faecal consistency was registered up to the end of the trial; in the ETEC F4 trial, the faecal consistency returned to normal within 3–4 days. No significant differences were found related to the synbiotic treatment.

Regarding the consistency of the ileal and colonic digesta, the oral challenge did not instigate significant changes in any of these parameters, although some effects were observed regarding the colon digesta’s consistency following the administration of the synbiotic. In the *Salmonella* trial, the synbiotic treatment was associated with improved consistency at day 8 PI (3.44 vs. 2.62; *p* = 0.010); moreover, in the ETEC F4 trial, it caused a trend towards interaction (*p* = 0.061) with an improvement with supplementation in challenged animals (3.62 vs. 3.00), but a looser consistency in the non-challenged group (3.87 vs. 3.62).

### Microbiological Analysis

**Figure 2:**
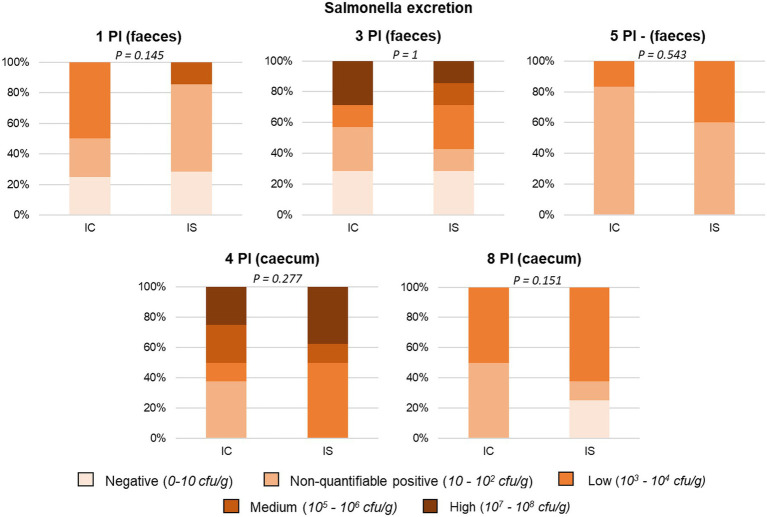
Percentage of animals included in the different faecal and caecal excretion levels of *Salmonella*. All samples were obtained from the heaviest animal in each pen, except for the caecal sample on day 4 PI, which was obtained from the anima of medium weight. IC, inoculated animals receiving placebo; IS, Inoculated animals receiving the synbiotic; and *N* = 8 for IC and IS. Values of *p* were obtained using Fisher’s Exact Test on R software.

In the *Salmonella* trial, the serological analysis revealed that all the animals remained seronegative throughout the study, confirming that they had not been exposed to the pathogen prior to the oral challenge. Regarding the presence of the pathogen in faeces and intestinal digesta, Figure 2 shows the evolution of *Salmonella* plate counts along sampling days in the challenged animals. In general terms, the non-challenged piglets remained negative during the study, with the exception of three piglets that recorded a positive result in at least one sample, albeit always at low-to-uncountable levels (< 10^2^ cfu/g).

All challenged animals were positive in at least one faecal sample and all presented *Salmonella* in the caecal content.

Regarding synbiotic administration, only a trend on day 1 PI was found, with the faeces revealing that the animals that received the synbiotic presented lower shedding compared to the control group (*p* = 0.145). Moreover, regarding the caecal digesta, 25% of piglets treated with the synbiotic turned negative to *Salmonella* excretion (vs. 0% control) on day 8 PI (P positive/negative excretion = 0.076).

Regarding the ETEC F4 trial, no differences between groups were registered in the plate counts of faecal enterobacteria or coliforms on the day of the piglets’ arrival. Furthermore, no changes caused by the synbiotic treatment were observed before the oral challenge, although differences could be noted related to the box (9.79, 10.69, 8.12 and 8.44 log cfu/g FM for IC, IS, NIC and NIS, respectively; *p* < 0.001). Table 3 shows the impact of the experimental treatments on the enterobacteria and coliform plate counts post-inoculation. The oral challenge promoted an increase in enterobacteria and coliforms either in the faeces (*p* < 0.013) or the ileal scrapings (*p* < 0.013) on day 3 PI. The effects produced by the challenge were no longer detected at day 7 PI. Moreover, no significant effects were found related to the synbiotic supplementation in any of the parameters analysed. However, a trend towards interaction in enterobacteria (*p* = 0.057) and coliform (*p* = 0.104) counts in the ileal scrapings could be identified at day 7 PI. On this day, non-challenged animals that received the synbiotic presented lower counts than the control, whereas challenged piglets exhibited the opposite effect.

**Table 3:** Effects of experimental treatments on enterobacteria and coliform counts in faecal samples and ileal scrapings.

	Treatment				RSD	*p*-value		
	IC	IS	NIC	NIS		Challenge	Treatment	Interaction
Enterobacteria (log cfu/g FM) Faeces								
Day 3 PI	10.44	10.51	9.15	8.83	1.564	0.012^*^	*0.828*	*0.725*
Day 7 PI	8.52	8.07	8.15	8.62	2.581	*0.925*	*0.993*	*0.615*
Ileal scrapings								
Day 3 PI	8.56	8.37	6.57	7.06	*1.564*	0.012^*^	*0.828*	*0.726*
Day 7 PI	7.08^y^	8.53^xy^	9.25^x^	7.98^xy^	*1.942*	0.247	*0.892*	*0.057*
Total Coliforms (log cfu/g FM) Faeces								
Day 3 PI	9.60	10.36	8.66	8.26	1.630	0.013^*^	*0.753*	*0.323*
Day 8 PI	7.90	7.51	8.10	8.55	2.446	*0.480*	*0.974*	*0.632*
Ileal scrapings								
Day 3 PI	8.30	8.07	6.25	6.95	*1.630*	0.013^*^	*0.753*	*0.323*
Day 7 PI	6.70	8.55	8.21	7.86	*1.850*	*0.532*	*0.263*	*0.104*

IC, Inoculated animals receiving placebo; IS, Inoculated animals receiving the synbiotic; NIC, Non-inoculated animals receiving placebo; and NIS, Non-inoculated animals receiving the synbiotic. *N* = 8 for all experimental groups. Values of *p* were obtained by ANOVA using the generalised linear procedure in R software. Letters x and y express differences considered for *p* < 0.07. RSD, residual standard deviation. *indicates statistical difference.

**Figure 3:**
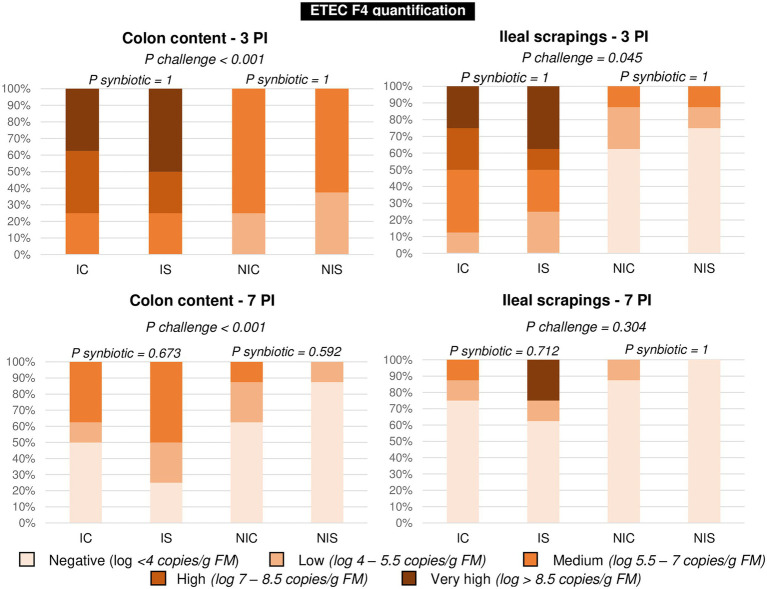
Percentage of animals in each presence level of ETEC F4 on days 3 and 7 post-inoculation. Different animals were sampled on day 3 PI (medium weight) and 7 PI (greatest weight). IC, inoculated animals receiving placebo; IS, Inoculated animals receiving the synbiotic; NIC, Non-inoculated animals receiving placebo; and NIS, Non-inoculated animals receiving the synbiotic. *N* = 8 for all experimental groups. Values of *p* were obtained using Fisher’s Exact Test on R software.

The results corresponding to qPCR targeting the coding gen of the F4 fimbria of *E. coli* F4 are summarised in Figure 3. Given that the pathogen could not be quantified in all animals, the data were analysed as frequencies. The figure shows the distribution of the percentage of animals within each of the five defined ranges based on the number of copies/g fresh matter found in the analysis. The effect of the oral challenge was clearly evidenced on day 3 PI through a significant increase in the percentage of animals showing large or very large numbers of copies in colonic content (*p* < 0.001) or ileal scrapings (*p* = 0.045). No significant differences were found related to the synbiotic administration.

### Intestinal Fermentation

Tables 4 and 5 show all the changes induced by the different experimental treatments in the ileal and colonic fermentation products.

**Table 4:** Effects of experimental treatments on ileal and colonic fermentation in the *Salmonella* trial.

	Day PI	Treatment				RSD	*p*-value		
		IC	IS	NIC	NIS		Challenge	Treatment	Interaction
*Salmonella* trial									
ILEUM									
Lactic acid (*mmol/kg*)	4	16.56	17.09	7.07	9.52	*20.010*	*0.341*	*0.911*	*0.913*
	8	22.38	6.01	7.37	3.10	*14.260*	*0.173*	*0.064*	*0.351*
Acetic acid (*mmol/kg*)	4	4.43	2.40	3.34	2.06	*2.301*	*0.479*	*0.077*	*0.711*
	8	2.95	2.97	2.37	2.40	*2.441*	*0.599*	*0.984*	*0.999*
COLON									
pH	4	6.00	5.89	6.20	6.03	*0.547*	*0.475*	*0.559*	*0.900*
	8	6.03	6.21	6.02	5.67	*0.335*	*0.070*	*0.986*	*0.819*
NH_3_ (*mmol/L*)	4	7.99	6.66	11.56	7.44	*3.877*	*0.210*	*0.169*	*0.414*
	8	30.75	38.80	15.39	13.57	*12.620*	0.001^*^	*0.366*	*0.377*
Lactic acid (*mmol/kg*)	4	6.20	1.80	0.24	1.11	*6.984*	*0.571*	*0.437*	*0.628*
	8	5.76	5.72	23.46	0.24	*14.340*	*0.376*	*0.272*	*0.155*
SCFA (*mmol/kg*)	4	93.1	89.6	107.9	97.1	*40.90*	*0.537*	*0.725*	*0.839*
	8	129.3	97.4	96.9	105.6	*45.16*	*0.543*	*0.332*	*0.313*
SCFA molar ratio (%)									
Acetic	4	53.8	54.3	57.8	54.8	*9.51*	*0.589*	*0.873*	*0.673*
	8	55.5^ab^	61.3^ab^	64.4^a^	45.3^b^	*10.50*	*0.437*	*0.571*	0.012^*^
Propionic	4	25.2	24.5	26.2	25.5	*6.03*	*0.695*	*0.777*	*0.998*
	8	28.5^b^	22.1^c^	24.5^c^	36.4^a^	*6.22*	*0.069*	*0.891*	0.002^*^
Butyric	4	13.1	13.1	11.9	14.0	*4.79*	*0.944*	*0.706*	*0.619*
	8	11.7	10.8	8.8	9.6	*4.22*	*0.268*	*0.865*	*0.650*
Valeric	4	4.19	5.04	2.16	3.89	*1.714*	0.044^*^	*0.117*	*0.557*
	8	3.33^b^	4.36^b^	1.66^b^	8.21^a^	*2.607*	*0.345*	0.014^*^	0.023^*^
BCFA	4	1.74	1.81	1.43	1.28	*1.388*	*0.491*	*0.997*	*0.856*
	8	0.82	1.36	0.65	0.42	*0.335*	*0.088*	*0.343*	*0.223*

This table includes values corresponding to pH, ammonia concentration (NH_3_; mmol/L of FM), lactic acid (mmol/L of FM) and total short-chain fatty acids (SCFA; mmol/L of FM and molar ratio) analysed in samples of ileal and colonic digesta.

IC, Inoculated animals receiving placebo; IS, Inoculated animals receiving the synbiotic; NIC, Non-inoculated animals receiving placebo; and NIS, Non-inoculated animals receiving the synbiotic. *N* = 8 for all groups except for non-challenged animals, *N* = 4. Values of *p* were obtained by ANOVA using the generalised linear procedure in R software. Letters a,b,c indicate statical differences between groups. FM, Fresh matter; NH_3_, ammonia concentration; SFCA, short-chain fatty acids; BCFA, branched-chain fatty acids; and RSD, residual standard deviation. *indicates statistical difference.

**Table 5:** Effects of experimental treatments on ileal and colonic fermentation in ETEC F4 trial.

	Day PI	Treatment				RSD	*p*-value		
		IC	IS	NIC	NIS		Challenge	Treatment	Interaction
ETEC F4 trial									
ILEUM									
Lactic acid (*mmol/kg*)	3	7.55	10.58	54.33	10.12	*15.880*	0.013^*^	*0.116*	0.004^*^
	7	14.42	18.06	15.96	14.52	*15.330*	*0.854*	*0.840*	*0.643*
Acetic acid (*mmol/kg*)	3	2.45	2.31	2.13	5.57	*1.727*	0.034^*^	*0.092*	0.033^*^
	7	3.93	3.85	3.86	6.20	*3.704*	*0.390*	*0.395*	*0.362*
COLON									
pH	3	6.14	6.09	6.26	6.38	*0.383*	0.035^*^	*0.548*	*0.096*
	7	6.05	6.08	6.21	6.15	*0.332*	*0.363*	*0.894*	*0.699*
NH_3_ (*mmol/L*)	3	9.56	10.14	5.8	4.82	*7.008*	*0.097*	*0.906*	*0.767*
	7	2.17	2.04	2.44	1.22	*1.288*	*0.532*	*0.150*	*0.253*
Lactic acid (*mmol/kg*)	3	0.65	3.10	1.65	2.64	*2.858*	*0.940*	*0.140*	*0.575*
	7	0.44	0.72	0.25	0.69	*0.397*	*0.667*	*0.106*	*0.704*
SCFA (*mmol/kg*)	3	99.8	99.3	81.8	103.4	*40.60*	*0.716*	*0.517*	*0.489*
	7	132.6	126.1	115.2	107.2	*21.94*	0.026^*^	*0.359*	*0.921*
SCFA molar ratio (%)									
Acetic	3	60.4	54.4	60.9	57.7	*4.53*	*0.261*	0.012^*^	*0.444*
	7	61.0	55.5	58.1	56.3	*4.45*	*0.515*	0.029^*^	*0.241*
Propionic	3	22.6^ab^	25.6^a^	22.8^ab^	19.4^b^	*3.37*	0.016^*^	*0.982*	0.022^*^
	7	22.6	23.7	25.0	24.4	*3.44*	*0.217*	*0.846*	*0.485*
Butyric	3	11.9	13.5	11.8	15.5	*3.78*	*0.483*	*0.093*	*0.464*
	7	13.1	15.2	12.4	13.1	*2.67*	*0.154*	*0.140*	*0.459*
Valeric	3	2.82	4.07	2.05	4.62	*1.741*	*0.959*	0.010^*^	*0.336*
	7	1.88	3.74	2.52	4.40	*1.328*	*0.174*	<0.001^*^	*0.981*
BCFA	3	1.74	1.32	1.37	2.08	*0.893*	*0.469*	*0.729*	*0.117*
	7	1.31	1.69	1.92	1.64	*0.779*	*0.325*	*0.862*	*0.243*

This table includes values corresponding to pH, ammonia concentration (NH_3_; mmol/L of FM), lactic acid (mmol/kg of FM) and total short-chain fatty acids (SCFA; mmol/kg of FM and molar ratio) analysed in samples of ileal and colonic digesta.

IC, Inoculated animals receiving placebo; IS, Inoculated animals receiving the synbiotic; NIC, Non-inoculated animals receiving placebo; and NIS, Non-inoculated animals receiving the synbiotic. *N* = 8 for all groups except. Values of *p* were obtained by ANOVA using the generalised linear procedure in R software. Letters a and b indicate statical differences between groups. FM, Fresh Matter; NH_3_, ammonia concentration; SFCA, short-chain fatty acids; BCFA, branched-chain fatty acids; and RSD, residual standard deviation. *indicates statistical difference.

As displayed, the challenge with *Salmonella* stimulated a significant increase in ammonia levels in the colon (34.77 vs. 14.48 mmol/l; *p* = 0.001) on day 8 PI. It did not influence the total amount of SCFA or lactic acid, but challenged animals presented a higher molar percentage of valeric acid in the colon on day 4 PI (4.61 vs. 3.02%; *p* = 0.044) and also tended to have more branched-chain fatty acids on day 8 PI (1.09 vs. 0.53%; *p* = 0.088). Although the administration of the synbiotic did not modify the ammonia, lactic or SCFA concentrations, it promoted a higher molar percentage of valeric acid in the colon on day 8 PI (6.28 vs. 2.49%; *p* = 0.014) and provoked two interactions in the molar percentages of acetic (*p* = 0.012) and propionic acids (*p* = 0.002). Whereas in non-challenged piglets administration of the synbiotic mixture reduced the molar percentage of acetic acid and increased that of propionic acid, in the challenged animals, the effect was the opposite.

In the ETEC F4 trial, the effects of the challenge in terms of fermentative activity were more apparent. In the colon, the challenge was responsible for a drop in pH on day 3 PI (6.11 vs. 6.38, *p* = 0.035) and an increase in the total amount of SCFA on day 7 PI (129.2 vs. 111.3 mmol/kg; *p* = 0.026). The molar proportion of propionic was also increased on day 3 PI (24.10 vs. 21.10%; *p* = 0.016), especially in the synbiotic group (P interaction = 0.022).

Related to the effects of the synbiotic mixture on the fermentation parameters, an interaction was found in the ileum on day 4 PI regarding the concentration of acetic acid and lactic acid as the main products of fermentation. Acetic acid showed the greatest concentration in the non-challenged animals not receiving the synbiotic (P interaction = 0.033), whereas lactic acid presented the highest values in the non-challenged and non-supplemented animals (P interaction = 0.004). At the colonic level, no effects were found for the pH, total concentration of SCFA or lactic acid, but changes did occur in the profile of fermentation. The percentage of acetic acid fell on both days (56.05 vs. 60.65% for day 3 PI, *p* = 0.012 and 55.9 vs. 59.55% for day 7 PI, *p* = 0.029), in line with an increase in valeric acid (4.34 vs. 2.43% for day 3 PI, *p* = 0.010 and 4.07 vs. 2.20% for day 7 PI, *p* < 0.001) and a trend towards a higher butyric acid molar percentage (14.52 vs. 11.88% for day 3 PI, *p* = 0.093 and 14.22 vs. 12.78% for day 7 PI, *p* = 0.140).

### Immune Response

No significant differences related to the synbiotic treatment were found in the serum levels of TNF-α or Pig-MAP. However, changes were noted associated with the pathogen inoculation. Regarding TNF-α, animals challenged with ETEC F4 presented higher concentrations than non-challenged pigs on day 7 PI (58.9, 57.2, 48.1 and 43.1 pg./ml for IC, IS, NIC and NIS, respectively; *p* = 0.010) and a similar pattern was found after the *Salmonella* challenge, although in this case, the differences did not reach statistical significance (125, 135, 124 and 101 pg./ml for IC, IS, NIC and NIS, respectively, day 8 PI; *p* = 0.132).

**Figure 4:**
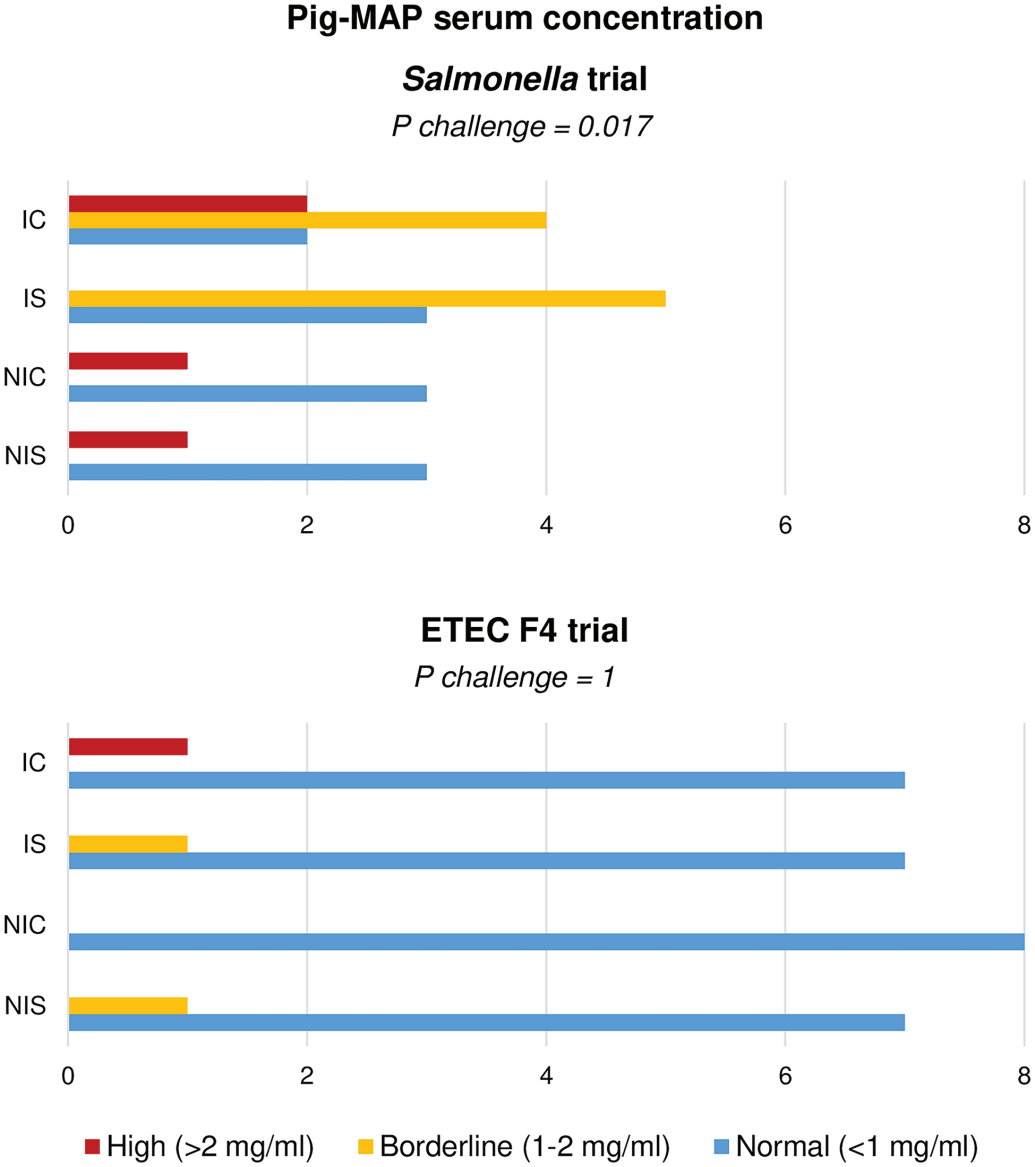
Effect of experimental treatments in serum levels of acute-phase protein Pig-MAP in piglets on day 8 (*Salmonella* trial) and 7 (ETEC F4 trial) following a pathogen oral challenge. Figure represents value frequencies between a normal (0.3–1 mg/ml) and abnormal (>2 mg/ml) range. IC, Inoculated animals receiving placebo; IS, Inoculated animals receiving the synbiotic; NIC, Non-inoculated animals receiving placebo; and NIS, Non-inoculated animals receiving the synbiotic. *N* = 8 for all experimental groups except for non-challenged animals in *Salmonella* trial, *N* = 4. Values of *p* were obtained using Fisher’s Exact Test on R software.

When analysing the Pig-MAP, the values did not adjust to a normal distribution and therefore the results were analysed as frequencies using Fisher’s exact test. Three range levels were defined as: high (> 2 mg/ml); borderline (1–2 mg/ml) and normal (< 1 mg/ml) according to Piñeiro et al. (2009). The results analysed in this way are shown in Figure 4. Only the *Salmonella* challenge was able to promote an increase in the number of animals with borderline-high levels of Pig-MAP at day 8 PI (*p* = 0.017), with no significant change induced by the ETEC F4 inoculation.

### Intestinal Histological Structure

The effects of the experimental treatments on ileal histomorphology are summarised in Table 6. The *Salmonella* challenge promoted shorter villi on day 4 PI (*p* < 0.001) and deeper crypts on days 4 and 8 PI (*p* = 0.013 and *p* = 0.042, respectively).

**Table 6:** Effects of treatments on histomorphological parameters on days 4 and 8 (*Salmonella* trial) and 3 and 7 (ETEC F4 trial) post-inoculation.

	PI Day	Treatment				RSD	*p*-value		
		IC	IS	NIC	NIS		Challenge	Treatment	Interaction
*Salmonella* trial									
Villi height (μm)	4	128	149	230	228	*50.4*	<0.001^*^	*0.532*	*0.604*
	8	215	202	227	270	*44.7*	*0.061*	*0.716*	*0.150*
Crypt depth (μm)	4	290	314	240	277	*24.4*	0.013^*^	*0.076*	*0.665*
	8	327	327	282	296	*40.1*	0.042^*^	*0.769*	*0.705*
IEL (Cell no./100 μm)	4	1.22	1.28	1.19	0.69	*0.636*	*0.276*	*0.626*	*0.320*
	8	1.01	1.41	1.14	1.53	*0.443*	*0.510*	0.039^*^	*0.994*
Mitosis (Cell no./100 μm)	4	0.90	0.93	0.60	0.85	*0.136*	*0.171*	*0.598*	*0.310*
	8	0.84	0.59	0.63	0.60	*0.408*	*0.142*	0.008^*^	*0.094*
ETEC F4 trial									
Villi height (μm)	3	256^ab^	267^ab^	287^b^	216^a^	*50.8*	*0.584*	*0.106*	0.032^*^
	7	285	261	261	294	*55.1*	*0.830*	*0.825*	*0.156*
Crypt depth (μm)	3	244	231	249	201	*47.8*	*0.475*	*0.081*	*0.308*
	7	223	219	217	245	*24.8*	*0.264*	*0.178*	*0.068*
IEL (Cell no./100 μm)	3	0.57	0.45	0.43	0.62	*0.252*	*0.871*	*0.705*	*0.086*
	7	0.49	0.40	0.45	0.43	*0.131*	*0.885*	*0.243*	*0.452*
Mitosis (Cell no./100 μm)	3	0.29	0.33	0.27	0.40	*0.084*	*0.546*	0.006^*^	*0.173*
	7	0.43	0.38	0.33	0.32	*0.091*	0.014^*^	*0.332*	*0.483*

IC, Inoculated animals receiving placebo; IS, Inoculated animals receiving the synbiotic; NIC, Non-inoculated animals receiving placebo; and NIS, Non-inoculated animals receiving the synbiotic. *N* = 8 for all groups except for non-challenged animals in Salmonella trial, *N* = 4. Values of *p* were obtained by ANOVA using the generalised linear procedure in R software. Letters a and b express differences considered for *p* < 0.05. IEL, villous intraepithelial lymphocytes; RSD, residual standard deviation. *indicates statistical difference.

After the ETEC F4 challenge, no significant differences were found apart from a greater number of mitosis on day 7 PI (*p* = 0.014).

Regarding impact of synbiotic administration on ileal histomorphology, different outcomes were found depending on the trial. In the ETEC F4 trial, villous height was greater on day 3 PI in the animals receiving the synbiotic, but only in the non-challenge group (P interaction = 0.032). However, the opposite numerical effect was observed on the second sampling day in both trials (P interaction <0.16). Crypt depth showed an increasing trend due to the synbiotic administration on day 4 PI in the *Salmonella* trial (*p* = 0.078), but, contrarily, a decreasing one on day 3 in the ETEC F4 trial (*p* = 0.081). IEL presence was enhanced by the synbiotic at day 8 PI in the *Salmonella* trial (*p* = 0.039) and the same trend was observed on day 3 PI in the ETEC F4 trial, although only in the non-challenged animals (P interaction = 0.086). Mitosis were reduced by the synbiotic at day 8 PI in the *Salmonella* trial (*p* = 0.008), especially in the challenged animals (P interaction = 0.094). In contrast, in the ETEC F4 trial, mitosis increased thanks to the synbiotic at day 3 PI (*p* = 0.006).

## Discussion

The aim of this work was to assess the potential of the combination of *B. longum* subsp. *infantis* CECT 7210 and a mixture of inulin and FOS as a synbiotic strategy to fight two common gastrointestinal pathogens: *Salmonella and* ETEC F4.

In our previous research (Barba-Vidal et al., 2017), the strain *B. longum* subsp. *infantis* CECT 7210 was found to have an effect against *Salmonella* Typhimurium and ETEC F4 colonisation in pigs with a stimulation of local immune response by increasing the number of intraepithelial lymphocytes. Furthermore, it is widely acknowledged that fructo-oligosaccharides (FOS) and inulin are selectively fermented by most strains of bifidobacteria (Wang and Gibson, 1993; Kaplan and Hutkins, 2000) due to the production of β-fructofuranosidases (Imamura et al., 1994). In fact, FOS and inulin are two of the most studied prebiotics with bifidogenic properties (Meyer and Stasse-Wolthuis, 2009). Considering these facts, we hypothesised that combining *B. infantis* CECT 7210 with FOS and inulin could therefore enhance its beneficial effects against pathogens, contributing to improved gut health.

With this objective, two different trials were performed to challenge animals with either *Salmonella* or ETEC F4. As is well known, these pathogens exhibit differences in terms of pathogenicity, mediated by distinct virulence factors and mechanisms (Boyen et al., 2008; Clements et al., 2012). Thus, the animals’ responses to the challenge and the clinical course differed depending on the pathogen. Whereas after the *Salmonella* challenge growth and feed intake were markedly reduced, with a clear effect on faecal score and an increase in rectal temperature, the challenge with ETEC F4 exhibited a much milder course of diarrhoea, the differences not being statistically significant for several parameters. Similar effects were observed by Barba-Vidal et al. (2017), viewing the challenge by ETEC F4 as milder than that of *Salmonella* Typhimurium.

The performance of the animals before the oral challenge was not modified by the administration of the synbiotic in terms of feed intake or weight gain, but in terms of gain:feed, a significant reduction in feed efficiency was seen in the *Salmonella* trial (0.48 vs. 0.66 for synbiotic vs. control diet, *p* = 0.02). We might hypothesise that this decrease in feed efficiency was the result of a dilution of the energy or limiting of amino acids in the SYN diet following the inclusion of 5% of the prebiotic. It may also have owed to changes in the transit time and digestibility of nutrients related to the inclusion of 5% of FOS/inulin (Kelly, 2009; Chen et al., 2017), being the changes that we observed in the consistency of caecal digesta at day 8 PI supported by this hypothesis. Furthermore, the reduction of gain:feed may have been due to modifications in the gut microbiota promoted by the synbiotic with an impact on host energy homeostasis (Rosenbaum et al., 2015; Ley et al., 2016). However, this is mere speculation, as we lack the evidence to support these ideas.

Clinical signs such as diarrhoea incidence were not improved by the administration of the synbiotic compound to the animals, but it is also fair to highlight that no deterioration was observed in any of the trials, verifying the safety of the probiotic strain, as proved by other authors (Moreno-Muñoz et al., 2011; Barba-Vidal et al., 2017; Rodríguez-Sorrento et al., 2020) even when it is combined with inulin and fructo-oligosaccharides.

Numerous previous studies have demonstrated that bifidobacteria, inulin and FOS can have a beneficial effect for the host, helping it to maintain a healthy gut environment. Indeed, bifidobacteria may increase the colonic intraluminal concentration of short-chain fatty acids (SCFA; Servin, 2004), which are responsible for a wide range of effects in the gastrointestinal system. Topping (1996) has described how the reduction in pH associated with an increase in SCFA might help to control the proliferation of pathogenic microorganisms. In addition, inulin and the fructo-oligosaccharides derived can produce the same effect on SCFA concentrations *in vivo* and *in vitro* (Pompei et al., 2008; Van der Beek et al., 2018; Baxter et al., 2019). In our trials, however, the concentration of SCFA in the colonic content was not enhanced by the synbiotic treatment. According to Nyman (2002), this outcome should be expected because the concentration of measured SCFA is contingent on the balance between production and absorption, and commonly SCFA produced by fermentation are rapidly absorbed or utilised by the colonic mucosa.

Regarding changes in the fermentation profile, different effects can be attributed to bifidobacteria and inulin or FOS. Several authors have described increases in the molar percentage of butyrate with probiotic bifidobacteria (Rossi et al., 2005; Belenguer et al., 2006), although the main fermentation product of bifidobacteria is acetate. Butyrogenic effects owe to a stimulation of acetate-depending, butyrate-producing colon bacteria by cross-feeding interactions that, in parallel, are required by some other bacteria that can convert lactate into butyrate, albeit only when acetate is present (De Vuyst and Leroy, 2011; Moens et al., 2016, 2017). Higher amounts of acetate and butyrate have been shown to have favourable effects on the colonic structure; for example, acetate promotes colonic epithelial proliferation and butyrate is responsible for the maintenance of mucosal integrity, reparation and colonocyte proliferation, given that it is the preferred energetic source for these cells (Topping, 1996). Inulin and FOS have been reported as exerting a bifidogenic effect in infants (Meyer and Stasse-Wolthuis, 2009; Paineau et al., 2013) as well as in modulating fermentation products in the gut. Furthermore, various *in vitro* studies have shown how inulin and oligofructose can increase butyrate, propionate (van de Wiele et al., 2007) and acetate (van der Beek et al., 2018) production. Differential effects may be related to the contrasting chemical structure of these compounds as well as to the specific microbial ecosystems in which they are introduced. In this regard, Rossi et al. (2005) has reported differences between inulin and FOS: whereas for inulin, the main fermentation product was butyric acid with lower amounts of acetic, lactic and propionic acids, for FOS, the main fermentation products were lactic acid and acetic acid, alongside lower amounts of butyric acid and no propionic acid. *In vivo* studies have also shown a variable impact on intestinal fermentation. Scholtens et al. (2006) have demonstrated increases in acetate and decreases in butyrate in the faeces of humans receiving 25–30 g/d of FOS for a period of 2 weeks, while Boets et al. (2015) have used stable isotope technology to demonstrate how in humans inulin is mainly fermented into acetate as well as to a lesser extent into butyrate and propionate.

Considering the varied findings of previous works, it was difficult to anticipate what to expect when combining bifidobacteria, inulin and FOS; moreover, the impact of the synbiotic would seem to be contingent on the trial in question. In the *Salmonella* trial, the synbiotic reduced the molar proportion of acetate in non-challenged animals but increased it in challenged ones. Differently, in the ETEC F4 trial, acetate was consistently reduced. These lower levels of acetate might be explained by a cross-feeding phenomena, supported in the ETEC F4 trial by the observable increasing trend of butyrate. In the same vein, the increase observed for acetate in the *Salmonella*-challenged animals with the synbiotic, could correspond to a more acute dysbiosis that might disturb the normal cross-feeding phenomena within bifidobacteria and colonic bacteria.

Similar kinds of interactions have been described in the literature. For instance, regarding FOS supplements to dogs, Pinna et al. (2018) found increases in the acetate:propionate ratio in low-protein diets but a decrease in high-protein diets.

Another SCFA that is rarely considered in the literature and whose concentration was augmented by the synbiotic treatment in both trials is valeric acid. This fatty acid, which is capable of inhibiting the growth of pathogenic bacteria like *Clostridium difficile* (McDonald et al., 2018), originates in 5-aminovalerate, which is a product of the anaerobic degradation of previously hydrolyzed protein by gut bacteria (Barker et al., 1987). Recent investigations have proved that a strain of *Megasphaera elsdenii* (a major inhabitant of the pig intestine) can utilise lactic acid as a fermentation substrate and convert it into valerate (Yoshikawa et al., 2018). It is feasible that a similar effect occurred in our experiments, as colonic lactic acid was augmented by the synbiotic, albeit only in the ETEC F4 trial.

Together with the inhibitory effects promoted by probiotics on enteropathogens throughout changes in the fermentation products, probiotics have also been shown to fight pathogens *via* other mechanisms. In particular, several species of *Bifidobacterium* are deemed capable of enhancing and modulating the immune response (Wagner et al., 2009; Bermudez-Brito et al., 2013; Presti et al., 2015), of releasing bacteriocins and bacteriocin-like substances (Poltavska and Kovalenko, 2012; Martinez et al., 2013) and of completing/displacing pathogens from their adhesion sites on the intestinal epithelium (Collado et al., 2005; Candela et al., 2008). The probiotic strain tested—*B. longum* subsp. *infantis* CECT 7210—has proven to be effective in reducing *Salmonella* loads in piglets in previous works (Barba-Vidal et al., 2017). Interestingly, the former strain administered together with *L. rhamnosus* HN001 was also capable of diminishing *Salmonella* and ETEC F4 presence as shown by our research group in the past (Rodríguez-Sorrento et al., 2020, 2021). Nevertheless, in the present trial, combining this probiotic with inulin and FOS was unable to reduce *Salmonella* excretion, suggesting that the combination of this *Bifidobacterium* strain with these prebiotics does not improve its power to fight the pathogen. Regarding the potential of *B. longum subsp. infantis CECT 72*10 to exclude ETEC F4, previous studies have demonstrated this strain’s ability to reduce the number of coliforms adhered to ileal mucosa in ETEC F4-challenged animals (Barba-Vidal et al., 2017), but in the present study, we were only able to find such an effect in the non-challenged animals. When challenged with the ETEC F4, the synbiotic treatment was associated with a numerical increase in attached coliforms, although not significantly. This interaction (day 7 PI; *p* = 0.057) may be explained by inoculated ETEC F4 potentially profiting from the supplemented inulin and FOS, considering that as stated by Rossi et al. (2005) *E. coli* can use these fermentable sources of carbohydrates as growth substrates, increasing its concentration when it is seeded in faecal cultures supplemented with inulin or FOS. However, despite these effects on coliforms, it is also important to remember that in both the present study and that of Barba-Vidal et al. (2017), no significant effects (nor increases or decreases) were detected in the numbers of ETEC F4 either in the digesta or in the ileal scrapings.

Although this investigation’s results do not provide evidence of the ability of the synbiotic to reduce the number of pathogens in the intestine, some insights regarding the potential positive effects can be provided. In the *Salmonella* trial, the reduction of villi height associated with the pathogen challenge (Olivares-Villagómez and Van Kaer, 2018) was not attenuated by the synbiotic mixture, although a decline in the number of mitosis was seen, suggesting that the amount of damaged tissue that needed to be replaced was reduced. Furthermore, the synbiotic may have also modulated the immune response at the gut level, as an increase in the number of IEL with the synbiotic was found in the *Salmonella* trial (*p* = 0.039) at day 8 PI as well as in the ETEC F4 trial, albeit only in those animals challenged with the pathogen (P interaction = 0.086). Similarly with this probiotic strain, Barba-Vidal et al. (2017) have also reported an increase in IEL in the ileum of piglets (whether challenged or not) with *Salmonella* of ETEC F4. A higher presence of IEL might be regarded as beneficial considering that these cells are responsible for the healing and protection of the integrity of the intestinal epithelium as well as acting as early response effectors against mucosal pathogens (Olivares-Villagómez and Van Kaer, 2018). Supporting the immunomodulatory properties of this strain, previous studies with a murine model of rotavirus infection (Moreno-Muñoz et al., 2011) have reported increases in the levels of secretory Immunoglobulin A (IgA) in the faeces. In the light of these results, increases in ileal IEL in the present study with the synbiotic could be attributed to the probiotic strain. However, a possible additional impact of the prebiotic fibres should not be discounted, considering that in the literature several works have reported the ability of inulin and FOS to enhance local immune responses (Shukla et al., 2016; Le Bourgot et al., 2017; Myhill et al., 2018).

To summarise, combining *B. longum* subsp. *infantis* CECT 7210 with inulin and FOS was not able to reduce *Salmonella* or coliforms loads in the gut, as has been previously reported for the single probiotic strain. Nonetheless, this synbiotic combination was able to modify the fermentative activity of the intestine with differential effects depending on the pathogen challenge, most likely disturbing the expected cross-feeding processes between the bifidobacteria and the indigenous butyrogenic colonic bacteria. The combination of this probiotic strain with inulin and FOS was also able to increase the numbers of IEL at the ileal level, suggesting certain immunomodulatory properties. A more in-depth study of the changes produced in the gut ecosystem is necessary in order to develop a greater understanding of the role of this synbiotic combination in a scenario of well-balanced or dysbiotic microbiota.

## Data Availability Statement

The original contributions presented in the study are included in the article/Supplementary Material, further inquiries can be directed to the corresponding author.

## Ethics Statement

The animal study was reviewed and approved by the Animal and Human Experimental Ethical Committee of the Autonomous University of Barcelona (Permit No. CEEAH: 4026; DMAH: 10118).

## Author Contributions

AR-S participated in the experimental design and was responsible for the animal trial, laboratory analysis, data analysis, and writing. LC participated in the experimental design, animal trials, data analysis, and writing. PL-C participated in animal trials and data analysis. GC-O and JM-M participated in the experimental design and contributed to data analysis and writing. SM-O participated in the experimental design, animal trials, laboratory analysis, data analysis, and writing. All authors contributed to the article and approved the submitted version.

## Funding

This project has been supported by CDTI (Ministerio de Ciencia e Innovación de España) and Fondo Europeo de Desarrollo Regional (FEDER; Acronym: SMARTFOODS, project number IDI-20141206).

## Conflict of Interest

GC-O and JM-M were employed by Laboratorios Ordesa SL.

The remaining authors declare that the research was conducted in the absence of any commercial or financial relationships that could be construed as a potential conflict of interest.

The handling editor declared a past co-authorship with the authors.

## Publisher’s Note

All claims expressed in this article are solely those of the authors and do not necessarily represent those of their affiliated organizations, or those of the publisher, the editors and the reviewers. Any product that may be evaluated in this article, or claim that may be made by its manufacturer, is not guaranteed or endorsed by the publisher.
